# Waiting Time for Pulmonary Vein Isolation: A Single-Center Retrospective Cohort Study of Atrial Fibrillation Progression and Complications

**DOI:** 10.3390/medicina62020276

**Published:** 2026-01-28

**Authors:** Kaspars Kupics, Matīss Linde, Kristīne Jubele, Oskars Kalējs, Natālija Nikrus, Sandis Sakne, Daiņus Gilis, Georgijs Ņesterovičs, Maija Vikmane, Evija Kanačniece, Ieva Ansaberga, Everita Kupriša, Matīss Karantajers, Andrejs Ērglis

**Affiliations:** 1 Latvian Center of Cardiology, Pauls Stradiņš Clinical University Hospital, LV-1002 Riga, Latvia; kaspars.kupics@gmail.com (K.K.); kristine.jubele@inbox.lv (K.J.); okalejs@gmail.com (O.K.);; 2 Faculty of Medicine and Life Sciences, University of Latvia, LV-1586 Riga, Latvia; 3 Faculty of Medicine, Riga Stradiņš University, LV-1007 Riga, Latvia

**Keywords:** atrial fibrillation, pulmonary vein isolation, waiting time, atrial fibrillation progression, rhythm control, catheter ablation

## Abstract

*Background and Objectives*: Pulmonary vein isolation (PVI) is an established rhythm control strategy for atrial fibrillation (AF). In many healthcare systems, increasing demand and limited procedural capacity have resulted in prolonged waiting times. The primary aim of this study was to evaluate the association between waiting time for PVI and AF progression. Secondary aims were to assess the relationship between waiting time and AF-related complications, healthcare utilization, and clinical factors associated with higher risk of progression. *Materials and Methods*: We performed a single-center observational cohort study of patients on the waiting list for PVI at Pauls Stradiņš Clinical University Hospital between 2016 and 2023. *Results*: A total of 341 patients completed structured ambulatory follow-up to assess the complication and progression rates of AF. The mean age was 64.8 ± 10.5 years, 50.9% were male, and the median waiting time was 37.2 months (IQR 15.0–61.3). AF progression occurred in 25.7% (*n* = 88) of patients, with longer waiting time independently associated with progression (OR, 1.017 per month; 95% CI, 1.006–1.028; *p* < 0.05). Electrical cardioversion during the waiting period was associated with a lower likelihood of progression (OR, 0.32; *p* = 0.029), and Class IC antiarrhythmic therapy was associated with reduced risk of AF progression (HR 0.78; *p* = 0.013). During follow-up, 45.2% of patients were hospitalized for AF paroxysms, 29.6% underwent electrical cardioversion, and 13.5% experienced complications including stroke and heart failure decompensation. Left atrial volume index and left ventricular ejection fraction were inversely correlated (ρ = −0.355, *p* < 0.05), but neither was associated with waiting time. *Conclusions*: Longer waiting times for PVI are associated with AF progression and substantial interim healthcare utilization due to complications. Strategies to prioritize higher-risk patients may help prevent disease progression and reduce complication burden.

## 1. Introduction

Atrial fibrillation (AF) is the most common sustained cardiac arrhythmia encountered in clinical practice and is associated with increased morbidity, mortality, and healthcare burden worldwide [1,2]. It has long been established that AF may progress from paroxysmal to persistent and ultimately permanent forms, often accompanied by a worsening of symptoms, reduced quality of life, and a heightened risk of stroke, heart failure, and hospitalization [3,4]. Pulmonary vein isolation (PVI) has become a cornerstone treatment for rhythm control in patients with symptomatic AF, particularly in those with paroxysmal or early persistent forms [5,6].

However, due to increasing patient demand and limited electrophysiology resources, prolonged waiting times for ablation procedures have become a growing concern in many healthcare systems [7,8]. While the efficacy of PVI in reducing arrhythmia burden and improving patient-reported outcomes has been well established [9,10], the impact of procedural delays on AF progression and adverse outcomes remains inadequately understood. It is hypothesized that prolonged delays may result in ongoing atrial remodeling and loss of sinus rhythm control, potentially reducing ablation success rates and increasing the incidence of complications such as stroke and heart failure [11,12].

Understanding the clinical consequences of extended wait times is crucial for optimizing patient prioritization and improving the allocation of healthcare resources. In this study, we aimed to evaluate the relationship between PVI waiting time and AF progression, hospitalization rates, and related complications in a real-world cohort. By investigating outcomes in patients awaiting PVI over an extended follow-up period, we hope to contribute data that may inform clinical decision-making and support timely intervention strategies for individuals with AF. Therefore, the objective of this study was to evaluate the association between PVI waiting time and AF progression, hospitalization frequency, and related complications in a real-world patient cohort and to identify clinical factors related to higher progression risk.

## 2. Materials and Methods

This was a single-center, retrospective observational cohort study conducted at Pauls Stradiņš Clinical University Hospital, Riga, Latvia. The study aimed to evaluate the impact of waiting time for PVI on AF progression, symptom burden, and complication rates.

Patients diagnosed with atrial fibrillation that were included on the PVI active waiting list between January 2016 and December 2023 were considered eligible. Patients were followed up in an ambulatory setting using a standardized structured questionnaire. We collected baseline and follow-up data on the following: AF characteristics—AF type (paroxysmal, persistent, permanent) at waiting list inclusion and follow-up and symptom severity according to the EHRA classification; clinical history and events—CHA_2_DS_2_-VASc score, frequency of hospitalization (AF paroxysm, stroke, heart failure decompensation, thromboembolism, syncope, bleeding), and number of electrical cardioversions; pharmacotherapy—current and prior antiarrhythmic drug use (Classes I–IV) and anticoagulation therapy; echocardiographic parameters—left atrial volume index (LAVI) and left ventricular ejection fraction (LVEF); and eligibility and prioritization for PVI—reassessment of indication, reasons for withdrawal, and criteria for expedited scheduling. The questionnaire enabled standardized longitudinal monitoring of disease progression, treatment response, and prioritization needs in a real-world AF cohort. Patients who were ultimately not scheduled for PVI were retained in the analysis because the study aimed to evaluate the clinical consequences of waiting for the procedure; progression to permanent AF or a decision to forgo ablation represents an outcome of the waiting period rather than a reason for exclusion.

Baseline characteristics were summarized using descriptive statistics. Categorical variables are presented as frequencies and percentages, and continuous variables as mean ± standard deviation (SD) or median with interquartile range (IQR), as appropriate. Group comparisons were performed using the χ^2^ test for categorical variables and the Mann–Whitney U test or Student’s t-test for continuous variables. Associations between waiting time and echocardiographic parameters were assessed using Spearman correlation analysis. Independent predictors of atrial fibrillation (AF) progression were identified using multivariable binary logistic regression, with results reported as odds ratios (ORs) and 95% confidence intervals (CIs). Time to AF progression was analyzed using Kaplan–Meier survival analysis, with comparisons between waiting time quartiles performed using the log-rank test. In addition, multivariable Cox proportional hazard regression was used to assess independent predictors of AF progression over time, with results reported as hazard ratios (HRs) and 95% CIs. All statistical tests were two-sided, and a *p*-value < 0.05 was considered statistically significant. Statistical analyses were performed using IBM SPSS Statistics, version 29.0 (IBM Corp., Armonk, NY, USA).

## 3. Results

A total of 341 patients were included in the final analysis. The mean age was 64.8 ± 10.5 years, and 50.9% (*n* = 174) were male. The average CHA_2_DS_2_-VASc score was 3.0 ± 1.8, and the median waiting time for pulmonary vein isolation (PVI) was 37.2 months (IQR: 15.0–61.3). Primary arterial hypertension 70.1% (*n* = 239), chronic heart failure 46.3% (*n* = 158), and coronary artery disease 27.0% (*n* = 92) were among the most prevalent comorbidities. Detailed population characteristics are represented in Table 1.

Over a median follow-up period of approximately 52 months, 25.7% (*n* = 88) of patients experienced progression of AF form—paroxysmal → persistent → permanent (Table 2). This included a significant reduction in paroxysmal AF (from 69.8% to 53.5%, *p* < 0.05) and an increase in persistent AF (from 30.2% to 32.0%, *p* < 0.05). In total, 14.3% (*n* = 49) of patients progressed to permanent atrial fibrillation and therefore were excluded from the PVI wait list. Patients with AF progression had a significantly longer median waiting time (52.2 months, IQR: 19.7–65.2) compared to those without progression (28.6 months, IQR: 14.3–56.1), *p* < 0.05.

In a binary logistic regression model adjusting for age, CHA_2_DS_2_-VASc score, prior AF-related hospitalization, electrical cardioversion during the waiting period, LAVI, and LVEF, waiting time remained an independent predictor of AF progression (OR, 1.017 per month; 95% CI, 1.01–1.13; *p* < 0.05). This corresponds to an approximately 22% increase in the odds of AF progression per additional year on the waiting list. No significant association was observed between wait time and CHA_2_DS_2_-VASc score, NYHA class, or comorbidity count (all *p* > 0.05), indicating that waiting time was primarily system-driven rather than patient-driven. Electrical cardioversion was independently associated with lower odds of AF progression (OR, 0.32; 95% CI, 0.11–0.89; *p* < 0.05). Age, CHA_2_DS_2_-VASc score, prior AF-related hospitalization, LAVI, and LVEF were not significantly associated with progression in the adjusted model (all *p* > 0.05). The model is depicted in Supplementary Table S2.

In the multivariable Cox regression including comorbidities, Class Ic antiarrhythmic drug (AAD) therapy remained independently associated with a lower risk of AF progression (HR 0.78; *p* = 0.013). Class III AADs did not predict progression when controlling for comorbidity burden (Supplementary Table S1).

**Figure 1:**
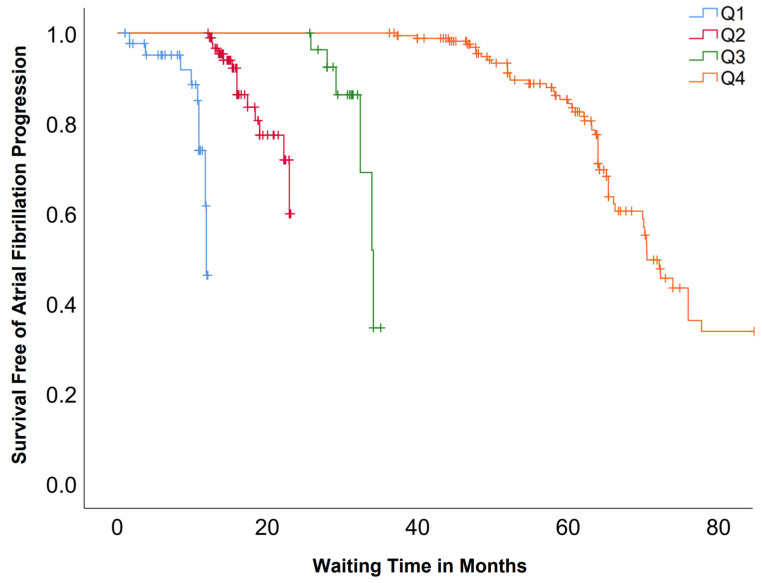
Kaplan–Meier curves of atrial fibrillation (AF) progression by waiting time quartiles. Q1 = 0 to 12 months; Q2 = 12 to 36 months; Q3 = 36 to 48 months; Q4 = above 48 months. Overall survival distributions differed significantly between groups (log-rank *p* < 0.05).

Kaplan–Meier survival analysis demonstrated marked differences in the progression of AF according to waiting time quartiles (Figure 1). Median progression-free survival was 11.8 months (95% CI, 11.1–11.8) for Q1, 21.3 months (95% CI, 20.5–22.2) for Q2, 34.0 months (95% CI, 32.1–35.9) for Q3, and 70.4 months (95% CI, 66.7–74.0) for Q4. The overall comparison was statistically significant (log-rank χ^2^ = 170.915, df = 3, *p* < 0.05).

Symptom burden (Table 2), assessed using the EHRA score, also changed significantly over time. The proportion of patients in EHRA class I increased from 11.2% (*n* = 38) to 26.7% (*n* = 91), while those in class IIb and III decreased from 42.8% (*n* = 145) to 29.3% (*n* = 100) and from 25.1% (*n* = 85) to 20.8% (*n* = 71), respectively (all *p* < 0.05). An almost statistically significant trend toward higher symptom burden at follow-up was observed among patients with AF progression (χ^2^ (4) = 9.05, *p* = 0.06).

During the waiting period, a total of 606 hospitalizations were recorded due to AF paroxysm in 154 (45.2%) patients. A total of 341 synchronized electrical cardioversions were performed for 29.6% (*n* = 101) of patients. Additionally, 93.5% (*n* = 319) received antiarrhythmic drug therapy, including 64.2% (*n* = 219) treated with Class IC AADs. Of the 101 patients who underwent cardioversion, 59.4% (*n* = 60) received Class IC therapy and 39.6% (*n* = 40) received Class III therapy; only one patient undergoing electrical cardioversion was managed without any antiarrhythmic medication.

Ischemic strokes occurred in 2.1% (*n* = 7) and hemorrhagic stroke in 0.3% (*n* = 1) of patients. Additional complications included heart failure decompensation 2.1% (*n* = 7), syncope 1.5% (*n* = 5), bleeding 1.2% (*n* = 4), and pulmonary embolism 0.3% (*n* = 1). Complications rates are presented in Table 3.

Assessing echocardiographic data at follow-up, the LAVI was 37.9 ± 9.4 mL/m^2^ and the mean LVEF was 59.3 ± 6.2%. Spearman’s correlation analysis revealed a significant moderate negative association between LAVI and LVEF (ρ = –0.355, *p* < 0.05). However, no significant correlations were found between waiting time and either LAVI (ρ = 0.071, *p* = 0.212) or LVEF (ρ = 0.013, *p* = 0.823).

In total, 241 (70.7%) patients remained eligible for PVI. Among the 100 patients for whom PVI was not indicated, the primary reasons included rate control strategy (52.0%), asymptomatic AF (16.0%), and lack of AF episodes in the past year (26.0%). A subset of 59 patients was clinically prioritized due to symptom progression, heart failure deterioration, or failure of antiarrhythmic treatment (Table 4).

We are aware of 19 patients that died while on the wait list for PVI, mostly due to underlying malignancy.

## 4. Discussion

This study demonstrated a significant association between prolonged waiting time for PVI and AF progression, symptom burden, and clinical complications. Our findings align with the evidence supporting early rhythm control strategies and suggest that extended delays in ablation may compromise long-term outcomes.

A key finding is the AF progression rate of 25.7% over a median waiting period of approximately 52 months, with progression strongly associated with longer wait times, as also demonstrated in Kaplan–Meier analysis, where patients in the longest waiting time quartile experienced higher progression of AF. It is noticeable that synchronized electrical cardioversion was associated with lower odds of AF progression. A meta-analysis by Blum et al. found a pooled AF progression rate of 8.1 per 100 patient-years, translating to approximately 20–25% over three years, especially in patients with persistent AF [13]. Another recent meta-analysis demonstrated that PVI within one year of atrial fibrillation diagnosis was associated with a 23% relative reduction in arrhythmia recurrence risk compared with longer diagnosis-to-ablation intervals [14]. This supports the notion that minimizing delays to ablation could be a modifiable factor to optimize post-procedural outcomes and underscores the importance of addressing system-level barriers—such as limited electrophysiology resources—that contribute to prolonged wait times. Moreover, the increased proportion of patients transitioning to persistent or permanent AF in our study is clinically significant. Progressive forms of AF are known to respond less favorably to ablation and carry a higher risk of complications such as thromboembolism and heart failure. The EAST-AFNET 4 trial previously established that early rhythm control in newly diagnosed AF patients significantly reduced adverse cardiovascular outcomes, including stroke and mortality [9].

Importantly, our analysis also reinforces the role of AADs. In particular, Class Ic AADs were associated with a significantly lower AF progression rate, suggesting a potential bridge strategy to maintain sinus rhythm while awaiting PVI. These pharmacologic findings support recommendations in the ESC 2024 guidelines to initiate early rhythm control in symptomatic AF patients awaiting ablation [15]. Rhythm control strategies were individualized based on symptoms, comorbidities, contraindications, prior drug response, and patient preference; therefore, not all patients received cardioversion or AAD therapy. This heterogeneity introduces potential confounding by indication, which is acknowledged as a limitation. Importantly, the majority of patients (93.5%) received AAD therapy, indicating that absence of rhythm control was uncommon.

In addition to clinical progression, our findings underscore the considerable healthcare burden imposed by prolonged delays. During the waiting period, 45.2% of patients experienced hospitalization due to AF paroxysms, totaling 606 hospitalizations, and 29.6% underwent synchronized electrical cardioversion (*n* = 341 procedures). These interventions consume substantial healthcare resources, including emergency department access, inpatient monitoring, and procedural capacity, which might otherwise be allocated toward definitive rhythm control strategies such as PVI. Frequent acute care episodes not only impact hospital operations but also increase healthcare costs and patient morbidity. This observation is supported by broader healthcare data. Peigh et al. found that AF patients incurred approximately 22% higher annual medical costs compared to non-AF patients, with AF-specific treatments—including ablation, cardioversion, antiarrhythmic drug therapy, and anticoagulation—accounting for about 36–40% of the excess cost in persistent and permanent AF, and over 78% in paroxysmal AF [16]. In the U.S. Medicare population, AF is associated with hospitalization rates more than double those of non-AF patients (37.5% vs. 17.5%) and significantly higher mortality [17]. These findings emphasize that timely PVI could reduce acute care burden, streamline healthcare utilization, and optimize resource allocation. Although we quantified hospitalizations and cardioversions, no formal cost analysis was performed. Given the high interim healthcare utilization observed, future work using a structured cost-effectiveness model could quantify economic impact more precisely and estimate potential savings associated with earlier ablation.

The observed complications—particularly stroke (2.4%) and heart failure decompensation (2.1%)—further support concerns that deferred intervention may permit cumulative thromboembolic and hemodynamic consequences. Similarly, a meta-analysis by Chew et al. found that longer diagnosis to ablation times were linked to significantly higher rates of AF-related hospitalizations and cardiovascular events [11].

Guiding future research is emerging evidence that suggests that novel circulating biomarkers may help identify patients at risk of AF progression and complications. Lower serum butyrylcholinesterase activity has been associated with greater AF prevalence, likely reflecting systemic inflammation and cardiometabolic dysfunction that contribute to atrial remodeling [18]. Multimarker studies further demonstrate that panels including N-terminal pro-B-type natriuretic peptide (NT-proBNP), high-sensitivity troponin, interleukin-6 (IL-6), growth differentiation factor-15 (GDF-15), and D-dimer improve predictions of stroke, heart failure hospitalization, and bleeding beyond clinical scores [19]. An atrial-specific candidate, bone morphogenetic protein-10 (BMP10), has been linked to ischemic stroke and may represent a marker of advanced atrial cardiomyopathy [20]. In our cohort, AF progression during prolonged waiting for PVI was mainly associated with an absence of effective rhythm control; the incorporation of such biomarkers in future studies could help identify patients who would benefit from earlier intervention and prioritization for ablation.

While symptom burden (EHRA score) showed general improvement in our cohort, likely due to interim rate and rhythm control measures, a non-significant trend toward worsening symptoms among those who experienced AF progression highlights the subjective toll of delayed ablation. Other real-world data have also proven quality-of-life deterioration during prolonged procedural wait times, with improvement observed only after ablation—particularly when AF burden is reduced—and a parallel reduction in healthcare utilization such as cardiovascular hospitalizations [21].

Interestingly, despite increased LAVI and its inverse correlation with LVEF, we found no direct relationship between these echocardiographic parameters and waiting time. This could suggest that structural remodeling is multifactorial and not solely dependent on procedural delays. Still, such echocardiographic findings may guide clinicians in prioritizing cases for early ablation.

Beyond demonstrating the clinical consequences of prolonged waiting times, our findings highlight the need for system-level interventions to reduce procedural delays. Wait list progression and scheduling at our center are limited primarily by electrophysiology laboratory capacity and specialist availability. Procedural volume is capped annually by institutional budgeting, resulting in fixed ablation slots per year. Prioritization is granted to patients with symptom progression, repeated hospitalizations, or failure of rhythm control therapy (Table 4). Potential strategies include structured prioritization based on symptom burden, hospitalization frequency, and failure of medical therapy; protecting a minimum procedural capacity through dedicated funding or ring-fenced electrophysiology laboratory time; and extending elective activity beyond standard hours, such as evening or weekend ablation lists. In settings with limited public capacity, formal collaboration with regional or private centers may also help reduce waiting lists. Although we did not perform a formal cost analysis, the high rates of interim hospitalizations and cardioversions observed suggest that even modest reductions in waiting time could be offset by lower acute care utilization and complication-related costs.

The strength of our study lies in its real-world design, long-term follow-up, and integration of both clinical and patient-reported outcomes. However, there are some limitations. First, its retrospective, single-center design introduces inherent risks of selection bias, information bias, and residual confounding, and precludes causal inference. Data were collected from clinical records and patient questionnaires, which may be subject to incomplete documentation and recall bias. Second, referral and prioritization patterns may reflect local organizational practices and therefore limit generalizability to other healthcare systems. Third, although we adjusted for relevant clinical variables, unmeasured confounders may have influenced the observed associations. A further limitation is that post-ablation outcomes were not available for this cohort; therefore, we could not evaluate whether longer waiting time was associated with AF recurrence or complications after pulmonary vein isolation. Assessment of the relationship between pre-procedural delay and long-term post-PVI success would be clinically important and should be addressed in future prospective studies. Finally, we did not perform a formal cost-effectiveness or resource utilization analysis, which limits economic interpretation of the findings.

Timely PVI should be prioritized, especially for patients with paroxysmal AF, significant symptom burden, or structural heart disease risk factors. Enhanced triaging algorithms—incorporating symptom progression, AAD response, and echocardiographic indicators—could optimize patient outcomes.

## 5. Conclusions

Prolonged waiting time for PVI is associated with a higher likelihood of AF progression, increased symptom burden, and a greater incidence of complications including stroke, heart failure, repeated hospitalizations, and cardioversions. These delays also contribute to substantial healthcare resource utilization through repeated hospitalizations and emergency interventions. Our findings support the need for improved triaging strategies to identify high-risk patients who may benefit from expedited ablation. Timely intervention not only holds the potential to mitigate clinical deterioration but may also alleviate the economic and operational burden on healthcare systems. In addition to clinical prioritization, health system measures such as protected procedural capacity, structured triage, and inter-institutional collaboration should be considered to reduce waiting times and mitigate preventable disease progression.

## Figures and Tables

**Table 1:** Population characteristics.

Item	*N* = 341 (%, Mean ±, Median IQR)
Gender	
Male	174 (50.9)
Female	167 (48.8)
Age, years	64.8 ± 10.5
Body mass index	26.2 ± 4.9
Waiting time, months	37.2 (IQR: 15.0–61.3)
CHA_2_DS_2-_VASc	3 ± 1.8
Coronary artery disease	92 (27.0)
Myocardial infarction in history	17 (5.0)
Primary arterial hypertension	239 (70.1)
Chronic heart failure	158 (46.3)
NYHA I	70 (20.5)
NYHA II	87 (25.5)
NYHA III	1 (0.3)
NYHA IV	0
Chronic kidney disease	28 (8.2)
Diabetes mellitus	52 (15.3)
Stroke in history	21 (6.2)
Ischemic	18 (5.3)
Hemorrhagic	2 (0.6)
Persistent foramen ovale	3 (0.9)
Pulmonary embolism	3 (0.9)
Oncology in history	13 (3.8)
Thyroid disease	36 (10.6)
Hyperthyroidism	18 (5.3)
Hypothyroidism	18 (5.3)
Prior PVI	38 (11.1)
Implantable cardiac device	
Pacemaker	29 (8.5)
ICD	1 (0.3)
CRT-D	2 (0.6)

IQR—interquartile range; CHA_2_DS_2_-VASc—congestive heart failure, hypertension, age ≥75 years (2 points), diabetes mellitus, prior stroke/transient ischemic attack (2 points), vascular disease, age 65–74 years, female sex; NYHA—New York Heart Association functional class; PVI—pulmonary vein isolation; ICD—implantable cardioverter–defibrillator; CRT-D—cardiac resynchronization therapy with defibrillator.

**Table 2:** Progression of atrial fibrillation and EHRA score.

	Inclusion	Follow-Up	*p*-Value
Atrial Fibrillation Form			
Paroxysmal	238 (69.8)	183 (53.5)	<0.05
Persistent	103 (30.2)	109 (32.0)	<0.05
Permanent		49 (14.3)	-
EHRA Score			
1	38 (11.2)	91 (26.7)	<0.05
2a	70 (20.6)	78 (22.9)	<0.05
2b	145 (42.8)	100 (29.3)	<0.05
3	85 (25.1)	71 (20.8)	<0.05
4	1 (0.3)	1 (0.3)	-

EHRA—European Heart Rhythm Association.

**Table 3:** Complication rates during waiting time for pulmonary vein isolation.

Item	*N* = 341 (%)
Atrial fibrillation paroxysm	154 (45.2)
Total count	606
Synchronized electrical cardioversion	101 (29.6)
Total count	341
Chronic heart failure decompensation	7 (2.1)
Stroke	
Ischemic	7 (2.1)
Hemorrhagic	1 (0.3)
Pulmonary embolism	1 (0.3)
Syncope	5 (1.5)
Bleeding	4 (1.2)
Other	2 (0.6)

**Table 4:** Re-evaluation for pulmonary vein isolation.

Item	*N* = 341 (%)
PVI is indicated	241 (70.7)
PVI is not indicated	*N* = 100 (%)
Patient declined	4 (4.0)
Major increase in LAVI	2 (2.0)
Asymptomatic atrial fibrillation	16 (16.0)
Heart rate control strategy	52 (52.0)
Other (no AF episodes in the last year)	26 (26.0)
PVI re-scheduled with higher priority	*N* = 59 (%)
Heart failure progression due to AF	5 (8.4)
Frequent hospitalizations due to AF	16 (27.1)
Symptomatic AF (EHRA ≥ 3)	36 (61.0)
Antiarrhythmic drugs are ineffective or not tolerated/allergic	24 (40.1)
Other	3 (5.1)

PVI—pulmonary vein isolation; AF—atrial fibrillation; LAVI—left atrial volume index; EHRA—European Heart Rhythm Association symptom score.

## Data Availability

The data presented in this study are not publicly available due to privacy and ethical restrictions related to patient confidentiality.

## Supplementary Materials

The following supporting information can be downloaded at https://www.mdpi.com/article/10.3390/medicina62020276/s1: Table S1: Multivariable Cox regression analysis for atrial fibrillation progression; Table S2: Multivariable logistic regression analysis for atrial fibrillation progression.

## Abbreviations

The following abbreviations are used in this manuscript:

AAD	antiarrhythmic drug
AF	atrial fibrillation
CHA2DS2-VASc	Congestive heart failure, Hypertension, Age ≥ 75 (2 points), Diabetes, Stroke/TIA (2 points), Vascular disease, Age 65–74, Sex category (female)
CRT-D	cardiac resynchronization therapy defibrillator
EHRA	European Heart Rhythm Association
HR	hazard ratio
ICD	implantable cardioverter–defibrillator
IQR	interquartile range
LAVI	left atrial volume index
LVEF	left ventricular ejection fraction
NYHA	New York Heart Association
OR	odds ratio
PVI	pulmonary vein isolation
SD	standard deviation
