# Supplementary material for: Waiting Time for Pulmonary Vein Isolation: A Single-Center Retrospective Cohort Study of Atrial Fibrillation Progression and Complications
Source: Medicina (Kaunas). 2026 Jan 28;62(2):276. doi: 10.3390/medicina62020276 (PMC12943156; doi:10.3390/medicina62020276)
Supplement: Supplementary file 1 [file medicina-62-00276-s001.zip › medicina-4112040-supplementary.pdf]

Supplementary Table S1. Multivariable Cox regression analysis for atrial fibrillation progression

Covariate	HR	95% CI	*p*-Value
Coronary artery disease	0.48	0.28–0.83	<0.01
Primary arterial hypertension	1.32	0.75–2.3	0.33
Chronic heart failure	1.57	1.18–2.09	<0.01
Diabetes mellitus	1.26	0.71–2.24	0.43
Valvular pathology	0.83	0.31–2.28	0.72
Thyroid disease	0.90	0.55–1.47	0.68
Chronic kidney disease	1.19	0.88–1.61	0.25
Oncology in history	0.47	0.11–2.02	0.31
Age (per year)	0.99	0.96–1.02	0.61
CHA_2DS_2-VASc (per point)	1.01	0.89–1.30	0.43
Class IC AAD	0.78	0.62–0.93	0.01
Class III AAD	0.94	0.74–1.20	0.63

HR—hazards ratio; CI—confidence interval; AF—atrial fibrillation; LAVI—left atrial volume index; LVEF—left ventricular ejection fraction; CHA_2DS_2-VASc—congestive heart failure, hypertension, age, diabetes, stroke, vascular disease, age, sex category score.

Supplementary Table S2. Multivariable logistic regression analysis for atrial fibrillation progression

Variable	OR	95% CI	*p*-Value
Age (per year)	0.99	0.95 – 1.03	0.73
CHA_2DS_2-VASc (per point)	1.21	0.96-1.5	0.12
Waiting time (per month)	0.017	01.01-1.13	< 0.05
Prior-AF related hospitalization	0.54	0.19-1.52	0.248
Electrical cardioversion during waiting period	0.32	0.11-0.89	0.03
LAVI (per ml/m^2)	1.09	0.978-1.09	0.14
LVEF (per %)	0.96	0.92-1.01	0.15

OR—odds ratio; CI—confidence interval; AF—atrial fibrillation; LAVI—left atrial volume index; LVEF—left ventricular ejection fraction.
